# Cytokines—Central Factors in Alcoholic Liver Disease

**Published:** 2003

**Authors:** Manuela G. Neuman

**Affiliations:** Manuela G. Neuman, Ph.D., is the director of the In Vitro Toxicology Laboratory in the Kunin-Lunenfeld Applied Research Unit, Baycrest Centre for Geriatric Care; and an assistant professor in the Department of Pharmacology and Institute of Drug Research, Faculty of Medicine, University of Toronto; both positions in Toronto, Canada

**Keywords:** cytokines, alcoholic liver disorder, biological activation, alcoholic hepatitis, fibrosis, liver cirrhosis, apoptosis, tumor necrosis factor-alpha, transforming growth factors, endotoxins, Kupffer cell, hepatocyte, disease course, disease susceptibility, immune system, chronic AODE (alcohol and other drug effects)

## Abstract

Many processes related to the consumption or breakdown of alcohol that contribute to alcohol-induced liver disease are mediated by small proteins known as cytokines, which are produced and secreted by liver cells and many other cells throughout the body. Through a variety of actions, cytokines regulate certain biochemical processes in the cells that produce them as well as in neighboring cells. For example, in case of an infection, they attract white blood cells to the tissues, triggering an inflammatory response. In the liver, persistent cytokine secretion resulting in chronic inflammation leads to conditions such as hepatitis, fibrosis, and cirrhosis. Cytokines also regulate a process known as programmed cell death, or apoptosis, which is in part responsible for alcohol-induced destruction of liver tissue. Two cytokines— tumor necrosis factor alpha and transforming growth factor beta—play prominent roles in apoptosis. Finally, a cytokine network mediates the harmful effects of a bacterial protein called endotoxin on the liver. Because of their diverse functions, cytokines might make attractive targets in the prevention or treatment of alcoholic liver disease, and researchers already have obtained encouraging results when testing such approaches.

Long-term excessive alcohol consumption can result in a spectrum of liver abnormalities, ranging from simple fatty liver (steatosis) or fatty liver accompanied by inflammation (steatohepatitis) to scar tissue formation (fibrosis), the destruction of the normal liver structure (cirrhosis), and even liver cancer (hepatocellular carcinoma). In its mildest form, fatty liver often causes no obvious clinical symptoms and is rarely fatal. In fact, only 15 to 20 percent of chronic heavy drinkers with steatosis have clinical liver disease, suggesting that other factors both in the drinker’s body (e.g., genetic influences) and in his or her environment help determine how alcoholic liver disease develops and progresses.

For liver damage to develop, numerous processes and biochemical reactions must occur in a variety of cells normally located in the liver or attracted to the liver when that organ is exposed to alcohol. This complex array of reactions is orchestrated by proteins called cytokines, which are produced and secreted by almost all cells in the body, including liver cells.

This article discusses the role of cytokines in alcoholic liver disease. A review of the general characteristics of cytokines is followed by an introduction to the process of programmed cell death, or apoptosis, which is regulated by cytokines and accounts for at least some of the liver damage found after chronic alcohol consumption. Finally, the article explores how a bacterial protein called endotoxin contributes to alcoholic liver damage by activating immune cells in the liver to release cytokines.

## Cytokines and Their Role in Cell Communication

Almost all cells in the body, including most types of liver cells, can produce and secrete cytokines. Released cytokines then interact with the cells whose functions they modify (i.e., the target cells). These target cells can be the same ones that initially produced the cytokines; this is called an autocrine effect. In addition, the cytokines can interact with neighboring cells; this is called a paracrine effect. Regardless of what the target cell of a cytokine is, the interaction occurs through a special docking molecule (i.e., a receptor) that consists of one or more proteins. This receptor has a specific three-dimensional shape, like a lock into which the cytokine “key” fits. The interaction between cytokine and receptor results in subtle alterations in the receptor’s structure, generating a communication signal that is conveyed into the cell’s interior, where it triggers a cascade of biochemical reactions, eventually altering the cell’s activities.

Most cytokines have more than one effect (i.e., are pleiotropic) and can influence more than one cell type. At the same time, many cytokines have overlapping actions. At least in some cases, these common effects result from the fact that the receptors for these cytokines share certain protein components and therefore mediate the actions of multiple cytokines. For example, the receptors for six different members of a group of cytokines called interleukins contain a protein called the cytokine receptor gamma chain. The specific receptors for each of these interleukins may be located on a different cell type, even within one organ; however, because all of these receptors contain the cytokine receptor gamma chain, their activation resulting from the binding of the various interleukins will have similar effects on the target cells.

Although cytokines have myriad effects throughout the body, their chief role is to help the body maintain a steady state. By releasing and responding to cytokines, cells protect the body against harm from foreign invaders (e.g., bacteria, viruses, and fungi) and from damage caused by internal toxic substances (e.g., alcohol and its breakdown products) and disease-causing cells (i.e., cancer cells).

### Cytokine-Producing Cells in the Liver

The liver consists of several cell types that under normal circumstances produce only minimal levels of cytokines (for a description of the various liver cell types, see the textbox). When the liver cells—particularly immune cells called Kupffer cells—become activated, however, cytokine production increases dramatically. If the liver has been damaged—for example, by trauma or excessive alcohol consumption—cytokines mediate the regeneration of liver tissue. Kupffer cells also can be activated by the presence of disease-causing micro-organisms or substances (i.e., pathogens). In this case, cytokines produced and released by the Kupffer cells induce an inflammatory response in the liver (i.e., hepatitis), which is required to start the healing process. (For a more detailed description of the immune system and inflammatory responses, see the sidebar.) However, if the inflammation does not subside after a short time, persistent production of these same cytokines may lead to scar tissue formation (i.e., fibrosis) and cirrhosis. Thus, cytokine production can have both beneficial and harmful effects, depending on the amount and duration of cytokine release.

**Liver Cell Types**

The liver contains numerous cell types, most prominently hepatocytes, endothelial cells, Kupffer cells, and stellate cells. *Hepatocytes* form the bulk (i.e., 80 percent) of the liver tissue. These cells are responsible for breaking down (i.e., metabolizing) molecules transported by the blood to the liver, including alcohol. *Endothelial cells* line the small blood vessels (i.e., sinusoids) that distribute blood throughout the liver. *Kupffer cells* belong to a group of immune cells called macrophages. Their main function is to ingest and destroy any foreign molecules or particles entering the liver (e.g., bacteria and bacterial proteins). *Stellate cells* have two distinct functions. When they are in a resting state, they serve to store fat in the liver. When they become activated (e.g., as a result of a liver injury), they produce proteins such as collagen that are required for tissue regeneration. Excessive activation of stellate cells leads to the formation of scar tissue, which is a characteristic of fibrosis.

**The Immune System and the Inflammatory Response**

The immune system, which protects the body against potentially disease-causing micro-organisms (pathogens) and other foreign molecules, consists of a vast array of immune cell types whose actions and responses to foreign molecules must be carefully orchestrated. Much of the coordination of immune system activity is carried out by molecules called cytokines, which are produced by certain immune cells; cytokines modify the activities of other immune cells. In general, the immune system can be divided into two arms, both of which involve several types of immune cells and require the actions of various cytokines:

- *Innate immunity*, which responds to any pathogen it encounters. Innate immunity exists even before the body is exposed to a pathogen for the first time.
- *Acquired immunity*, which amplifies the reaction of the innate immunity. Acquired immunity is activated only after the body is exposed to a given pathogen for the first time, and responses of the acquired immune system are specific to the particular pathogen. The activated cells of the acquired immunity also constitute an immune memory—they “remember” the pathogen to which they respond, giving them the ability to fight a second infection by that pathogen even faster and more efficiently.

One central component of innate immunity is a type of white blood cell that can ingest and thereby destroy foreign pathogens (i.e., phagocytes). Phagocytes include cells called neutrophils, which primarily ingest invading bacteria; natural killer cells, which eliminate cells that have been infected by parasites or have turned into cancer cells; and monocytes, which ingest a variety of foreign molecules and micro-organisms. Of these groups, monocytes are of particular interest to this discussion because they produce cytokines, which help regulate immune system activity. In addition, monocytes display proteins called antigens (derived from ingested pathogens and other molecules) on their surface, thereby activating cells involved in acquired immunity to further enhance the body’s response to the pathogen. Some monocytes stop circulating in the blood and enter the tissues; these cells, which then are called macrophages, have the same functions as circulating monocytes. The largest number of macrophages resides in the liver; these macrophages are called Kupffer cells.

The main components of acquired immunity are white blood cells called T-lymphocytes (T-cells) and B-lymphocytes (B-cells). T-cells circulating in the blood and lymph recognize and bind to monocytes or macrophages that display antigens on their surface. This interaction activates the T-cells, causing them to multiply and produce cytokines and chemokines. Chemokines attract additional immune cells to the site of the infection in an effort to destroy the infected cells and thereby contain the infection. B-cells also recognize and bind to foreign antigens. Subsequently, B-cells begin to produce and release large amounts of immune molecules called antibodies, which circulate throughout the blood and bind to those antigens (or to the bacteria from which the antigens were derived) wherever they encounter them. The antibody-covered antigens or bacteria then can be recognized and destroyed by monocytes.

— *Manuela G. Neuman*

Kupffer cells, which play an important role in inflammatory responses, as just described, are macrophages— immune cells that enter the tissues and destroy foreign pathogens and other harmful substances. For example, Kupffer cells eliminate bacteria and viruses that have entered the liver, as well as damaged or abnormal liver cells, and liver cells that have died by apoptosis (see the section “Apoptosis, Cytokines, and Alcoholic Liver Disease”) or as the result of an inflammatory response. Macrophages also secrete numerous cytokines that help coordinate the actions of other immune cells in response to pathogens and toxic compounds. Macrophages are found in all tissues, but the largest number reside in the liver.

Together with other immune cells, macrophages such as the Kupffer cells provide the body’s first line of defense— an acute inflammatory reaction— against bacteria and virus infections as well as other toxic substances, such as alcohol. In addition to ingesting and destroying pathogens, these cells secrete cytokines that attract other immune cells to the site of the infection. The increased blood flow that delivers those cells to the infection site, combined with other consequences of the infection, results in the typical symptoms of inflammation—pain, redness, and swelling. Under normal conditions, the levels of these inflammation-promoting (i.e., pro-inflammatory) cytokines and the resulting inflammatory response decrease once the infection is under control. If the levels of inflammatory cytokines remain elevated, however, a chronic inflammation ensues. This is the case in alcohol-induced chronic inflammation of the liver (i.e., alcoholic hepatitis), which can be a precursor to fibrosis and cirrhosis.

Based on their specific functions, inflammation-fighting cytokines fall into the following groups (for information on specific cytokines, see the table):

- *Pro-inflammatory cytokines* (e.g., interleukin[IL]–1, IL–6, tumor necrosis factor alpha [TNF–α], and transforming growth factor beta [TGF–β]), which stimulate the growth and development of various immune cells, activate macrophages to release more of the same cytokines, and induce the production of other molecules required for an inflammatory response.
- *Immunoregulatory cytokines* (e.g., IL–10), which help regulate the immune response by inhibiting the proliferation of certain immune cells and promoting the proliferation of others; reducing the production of inflammatory cytokines; and promoting the secretion of antibodies, which bind to specific foreign molecules, thereby inactivating those molecules and marking them for destruction by other immune cells.
- *Chemokines * (e.g., IL–8), which attract a certain type of immune cell (i.e., neutrophils) to the site of an infection.

Pro-inflammatory and immunoregulatory cytokines are discussed in more detail throughout this article. Chemokines initially had no known biologic activity; investigators only knew that they were associated with inflammatory diseases, such as alcoholic hepatitis. Only after IL–8 and other chemokines called monocyte chemoattractant protein 1 and macrophage inflammatory protein 1α and 1β all were found to attract white blood cells to the site of an infection did it become clear that these proteins share important structural and functional features. More recently, studies have demonstrated that liver cells involved in tissue regeneration (i.e., stellate cells) produce chemokine receptors and respond to the actions of chemokines such as IL–8 (Neuman et al. 2001).

### Alcohol’s Effects on the Immune System and Cytokines

Chronic alcohol use has adverse effects on the immune system, as shown by the fact that alcoholics have a higher incidence of infectious diseases and deficiencies of the immune system than nonalcoholics (Molina et al. 2002). For example, many alcoholics are infected with the hepatitis C virus or the human immunodeficiency virus (HIV). Both of these infections, particularly if they occur together, may interfere with the normal networks of cytokines, possibly increasing patients’ risk of liver cancer.

As indicated above, the liver is crucial to the body’s initial response to an infection—that is, to the inflammatory response—because it contains large numbers of macrophages (the Kupffer cells) that help detect toxic substances or micro-organisms in the blood and secrete cytokines to coordinate the inflammatory response. However, if cytokine levels remain persistently elevated in the liver (e.g., as a result of alcohol’s actions on the body), chronic inflammation of the liver (i.e., hepatitis) ensues. Clinical studies have demonstrated that patients with alcoholic liver disease have increased levels of the inflammatory cytokines IL–1, IL–6, and TNF–α as well as the chemokine IL–8 and other cytokines (McClain and Cohen 1989; McClain et al. 1997). These cytokines probably are responsible for at least some of the symptoms associated with alcoholic hepatitis, such as fever, metabolic changes, and weight loss.

The liver also is involved in subsequent steps of the immune response to many infections. For example, if the primary liver cells, the hepatocytes, become infected by a virus, they display pieces of viral proteins on their surface. These viral protein fragments attract immune cells called T-cells, which destroy virus-infected cells, to the liver. T-cells attach themselves to the infected hepatocytes through the interaction between a receptor on the T-cells and the viral protein pieces displayed together with other molecules on the surface of the hepatocyte. This interaction leads to the destruction of the infected hepatocyte.

Chronic alcohol consumption, however, has detrimental effects on the T-cells’ ability to destroy the infected cells. For example, alcoholics have lower-than-normal numbers of all types of T-cells (Szabo 1997). Moreover, alcohol may impede the T-cells’ ability to multiply and to exert their influence after they have been activated (Szabo 1997). As a result, the body cannot mount an effective immune response, rendering the alcoholic more susceptible to infections with pathogenic micro-organisms. In addition, an impaired immune response leads to increased apoptosis of both infected and noninfected cells, thereby contributing to liver damage.

## Apoptosis, Cytokines, and Alcoholic Liver Disease

Another way in which cytokines contribute to alcoholic liver disease is through programmed cell death, or apoptosis. Apoptosis is genetically determined; each cell in the body carries the genes necessary to initiate the processes leading to apoptosis. Under normal conditions, apoptosis helps ensure the correct functioning of the body’s cells, as described in the following sections. Excessive or inappropriate apoptosis, however, can lead to tissue damage, including alcoholic liver disease (Neuman 2001).

**Table:** Cytokines Involved in Alcoholic Liver Disease

Cytokine	Principal Function
*Pro-inflammatory cytokines*	
Interleukin–1 (IL–1)	Produces inflammatory responses; induces fever; stimulates growth and differentiation of the immune system
Interleukin–6 (IL–6)	Promotes maturation of antibody-secreting B cells; acts with other cytokines to stimulate other immune system cells; stimulates production of mediators of inflammatory responses; stimulates liver regeneration
Tumor necrosis factor alpha (TNF–α)	Promotes inflammatory responses; stimulates neutrophils and macrophages; induces fever; induces macrophages to produce cytokines; induces both apoptosis and necrosis
Transforming growth factor beta (TGF–β)	Promotes collagen synthesis
*Immunoregulatory cytokines*	
Interleukin–10 (IL–10)	Inhibits proliferation of certain immune system cells and promotes proliferation of others; reduces production of inflammatory cytokines; promotes antibody secretion
*Chemokines*	
Interleukin–8 (IL–8)	Attracts neutrophils to the site of an infection.

### A Brief Review of Apoptosis

For the body to function properly, it must ensure that damaged cells or cells that are no longer needed can be destroyed in a safe manner. This destruction is accomplished by apoptosis. All cells constantly survey their external and internal environments for signals promoting cell survival or cell death. For example, the presence of growth factors in the medium surrounding the cell indicates cell survival. Conversely, the presence of certain other molecules in the environment or the altered activities of certain genes in the cell are signals that promote cell death. Every cell monitors and integrates the sometimes-conflicting signals it receives to decide whether to live or commit suicide. When the “death signals” prevail, the cell initiates a series of biochemical reactions that result in its death.

**Figure 1:**
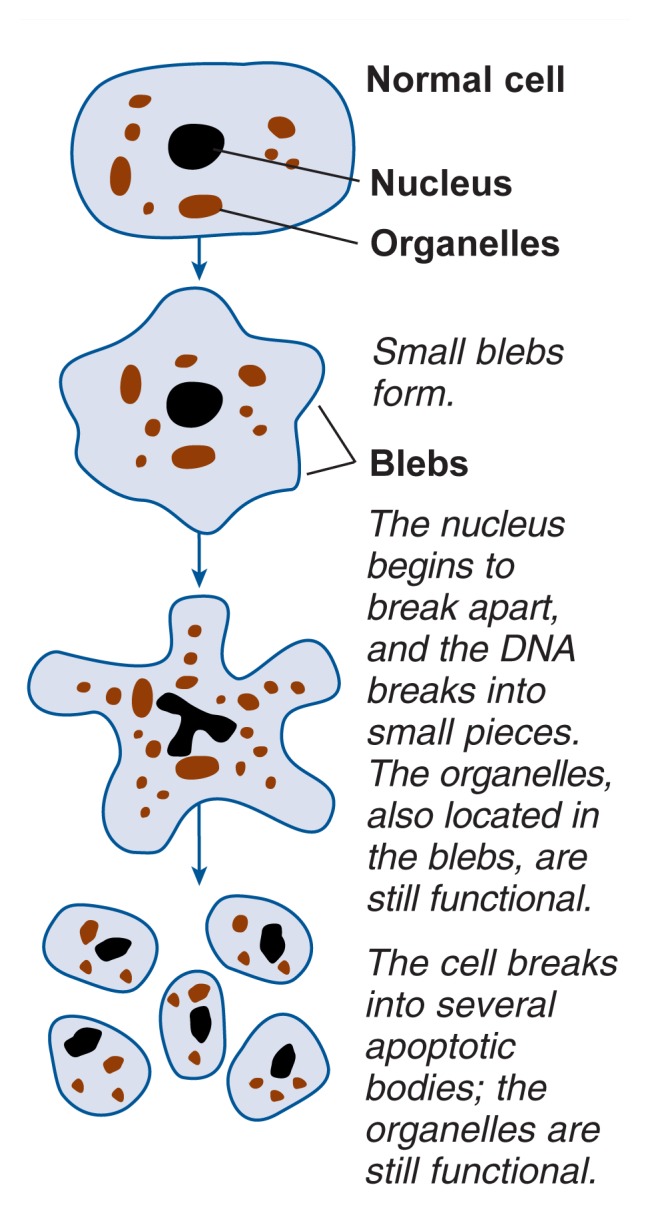
Schematic representation of the process of apoptosis.

To avoid putting additional stress on the organism, apoptosis proceeds so that no inflammatory reaction is initiated (see figure 1). When a cell undergoes apoptosis, it severs its contacts with neighboring cells and begins to form small protrusions (i.e., blebs). Next, the cell’s nucleus breaks apart and the DNA breaks into small pieces. These nuclear and DNA pieces, as well as other cell components (i.e., organelles) are distributed among the blebs, which increase in size. Each bleb eventually encloses a portion of the cell’s content, and the cell breaks apart, forming several so-called apoptotic bodies which can then be ingested and destroyed by macrophages and other cells. In this manner, none of the cell content is released to cause an inflammatory response.

In the liver, apoptosis may play an important role in eliminating hepatocytes that no longer are needed or whose DNA has been damaged. For example, under certain conditions the number of liver cells increases. When those excess cells are no longer needed, apoptosis probably helps decrease liver mass again. In general, hepatocytes seem to be particularly susceptible to apoptosis (Neuman 2001).

Several endogenous compounds such as cytokines, or foreign compounds such as alcohol and other drugs, can trigger hepatocyte apoptosis. A better understanding of the role that apoptosis plays in alcohol-induced liver diseases and of the mechanisms by which apoptosis occurs in the liver may give researchers insights into these diseases and point to possible treatments.

### Alcohol-Induced Apoptosis

**Figure 2:**
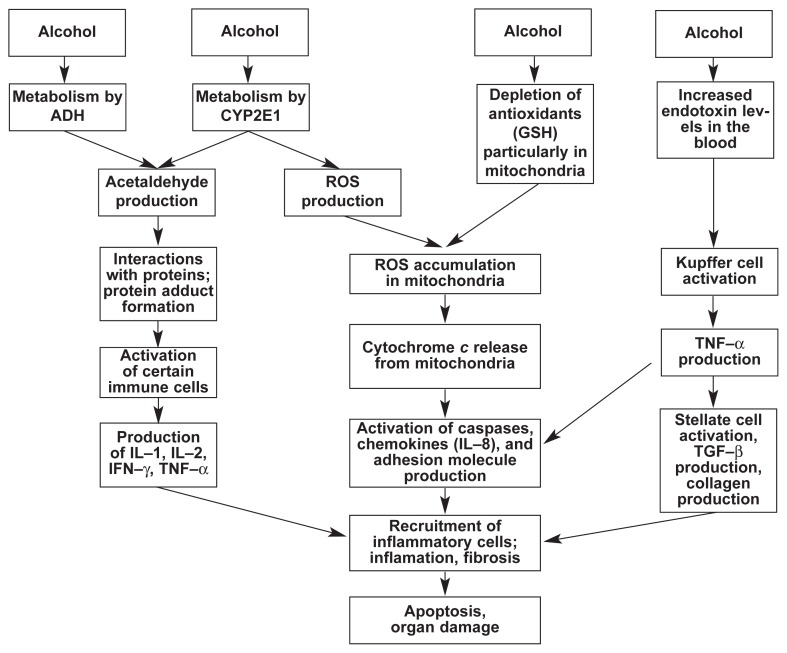
Pathways through which alcohol (ethanol) can contribute to apoptosis. Alcohol is broken down (i.e., metabolized) in the liver cells by two enzymes, alcohol dehydrogenase (ALD) and, particularly after chronic alcohol consumption, cytochrome P450 2E1 (CYP2E1). Both enzymes convert alcohol to acetaldehyde, a toxic substance. Some of the acetaldehyde interacts with proteins in the cells, forming compounds called adducts that can activate certain immune cells to produce various cytokines, including interleukins (ILs), interferon gamma (IFN–γ), and tumor necrosis factor alpha (TNF–α). In addition to acetaldehyde, alcohol metabolism by CYP2E1 also generates highly reactive molecules known as reactive oxygen species (ROS), which accumulate primarily in cell structures called mitochondria. ROS normally are eliminated from the cells by compounds known as antioxidants, particularly a small molecule called glutathione (GSH). Alcohol, however, depletes the cell’s GSH stores, thereby further exacerbating ROS accumulation in the mitochondria. This process leads to the release of cytochrome *c* from the mitochondria, which then activates enzymes called caspases and promotes production of IL–8 in the cell. Finally, alcohol leads to increased levels of a bacterial protein called endotoxin in the blood and in the liver, which activates immune cells called Kupffer cells that reside in the liver. These cells then produce TNF–α, which in turn activates another type of liver cell, the stellate cells, to produce transforming growth factor beta (TGF–β) and collagen, a protein involved in scar tissue formation (fibrosis). TNF–α production also leads to increased production of chemokines (e.g., IL–8), which attract inflammatory cells from the bloodstream to the liver, contributing to liver inflammation. Excess TNF–α and chemokine production also causes increased production of adhesion molecules that play an important role in fibrosis. Thus, all of these diverse pathways contribute to inflammatory reactions and fibrosis and culminate in the induction of apoptosis and organ damage.

Numerous factors related to alcohol and its breakdown, which occurs primarily in the liver, can induce apoptosis of hepatocytes. As described in other articles throughout this journal issue, the processes by which alcohol is broken down in the hepatocytes generate a variety of molecules that can be toxic to the liver or interfere with normal physiological processes (see figure 2). For example, alcohol breakdown through the enzyme known as cytochrome P450 2E1 (CYP2E1) leads to the formation of small oxygen-containing molecules called reactive oxygen species (ROS). These ROS, unless they are rapidly eliminated or converted into harmless molecules by antioxidants, can interact with and damage complex molecules in the cells (e.g., proteins and DNA). (For more information on ROS and their harmful effects, see the article in this issue by Wu and Cederbaum.) Both increases and decreases in the levels of ROS can lead to apoptosis of hepatocytes.

Alcohol and its breakdown not only result in the formation of ROS and other reactive molecules in the cell but also reduce the levels of certain antioxidants. Decreases in an important antioxidant, glutathione (GSH), in the cells have been shown to be an early event in apoptosis. GSH is found both in the fluid filling the cells (i.e., the cytosol) and in small cell components called mitochondria, in which most of the cell’s energy production occurs. For the cell to function normally, it is important that enough GSH is present in the mitochondria because most of the ROS are formed there. Unless these ROS are rapidly eliminated by GSH, they can damage the mitochondria, allowing the molecule cytochrome *c* to leak from the mitochondria into the cytosol. Once in the cytosol, cytochrome *c* can activate enzymes known as caspases that can trigger apoptosis.

Alcohol can reduce GSH levels in the cells, particularly in the mitochondria. For example, when isolated hepatocytes were exposed to alcohol, GSH levels in the mitochondria (but not in the cytosol) decreased dramatically (Neuman et al. 1998).^1^ Hirano and colleagues (1992) showed that GSH depletion is partly responsible for cytotoxicity caused by alcohol. Additional experimental studies have suggested that the depletion of GSH in the mitochondria could be a result of impaired GSH transport from the cytosol (Garcia-Ruiz et al. 1995*a*,*b*; Fernandez-Checa et al. 1996; Kaplowitz et al. 1996; Kaplowitz and Tsukamoto 1996; Neuman et al. 2002).

GSH depletion may be one of the factors contributing to alcohol-induced apoptosis. In addition, alcohol exposure led to the activation of caspase 3 and other signals triggering apoptosis. When alcohol-exposed hepatocytes were viewed under an electron microscope, damage to the mitochondria was the first indication that the cells were undergoing apoptosis (Katz et al. 2001). These hepatotoxic effects of alcohol and its breakdown products are amplified by responses from the immune system (e.g., activation of T-cells and release of pro-inflammatory cytokines).

Another element linked to hepatocyte apoptosis is a system consisting of two molecules, Fas and Fas ligand. Fas, also known as CD95, is a type of receptor found on hepatocytes and is similar to one of the receptors for the cytokine TNF–α (also discussed in the next section). This Fas receptor can interact with the Fas ligand, which is present on the surface of certain T-cells. The interaction between the Fas ligand and soluble Fas triggers chemical processes in the hepatocyte that lead to apoptosis. As a result of these processes, apoptosis-inducing caspases are activated, the mitochondria become leaky, and other apoptosis-promoting reactions are initiated. One of the factors that may promote binding of Fas and the Fas ligand is the presence of ROS—for example, those ROS generated by the breakdown of alcohol (Navder and Lieber 2002). Thus, ROS can adversely affect hepatocytes by damaging the cell’s DNA, promoting apoptosis induced by the Fas/Fas ligand system, and altering the activities of various other genes in the cells.

### Cytokines Regulating Apoptosis

Two cytokines—tumor necrosis factor alpha (TNF–α) and transforming growth factor beta (TGF–β)—strongly influence apoptotic processes, including apoptosis of hepatocytes.

### Tumor Necrosis Factor Alpha (TNF–α)

TNF–α is one of the inflammatory cytokines; among other functions it triggers the production of additional cytokines. In the liver, these cytokines together attract inflammatory cells to the organ, destroy hepatocytes, and initiate a healing response that includes the formation of scar tissue. The structure of TNF–α is similar to that of the Fas ligand; consequently, TNF–α shares some of the functions of Fas ligand, such as promoting apoptosis. For example, researchers have found that TNF–α can induce apoptosis in cells derived from a human liver tumor (i.e., hepatoma cells) (Neuman et al. 1998) as well as in hepatocytes obtained from mice and rats (Leist et al. 1997; Kurose et al. 1997*a*,*b*). TNF–α also may play a role in alcohol-induced apoptosis. For example, studies have found that whereas the liver (and other tissues) normally produces only minimal levels of TNF–α, the levels of that cytokine were substantially increased in the blood of alcoholic patients with acute or chronic liver disease (Pastorino and Hoek 2000).

The ultimate effect of TNF-α on hepatocytes in an animal or human is strongly influenced by the presence of other cytokines in liver tissue. In normal mice, TNF–α is essential for liver regeneration. If part of an animal’s liver is removed, TNF–α assists in liver regeneration by promoting the proliferation of hepatocytes. If the mice lack the pro-inflammatory cytokine IL–6, however, increased production of TNF–α resulting from partial removal of the liver promotes the death of hepatocytes (Tilg and Diehl 2000). Similarly, destruction of the gene for the regulatory cytokine IL–10 (which helps terminate inflammatory responses) exacerbates TNF-mediated liver injury in mice (Tilg and Diehl 2000). Conversely, mice that are deficient in several other cytokines—such as IL–18 and interferon-gamma, which promote TNF–α activation and, consequently, apoptosis— are protected against the apoptosis-promoting effects of TNF–α (Tilg and Diehl 2000).

#### Consequences of Alcohol Exposure on the Effects of TNF–α

Studies have found that alcohol may increase the liver’s sensitivity to inflammatory cytokines, such as TNF–α, in two ways. First, alcohol may directly or indirectly stimulate Kupffer cells to produce and release TNF–α into the small channels (i.e., sinusoids) in which the blood flows through the liver. (One indirect mechanism—the alcohol-induced increase in the levels of a bacterial endotoxin in the blood—is discussed in the next section.) Second, alcohol may enhance the sensitivity of hepatocytes to TNF–α (Nanji et al. 2001). TNF–α might increase the metabolism of the hepatocytes, particularly energy production in their mitochondria, which could lead to an increased production of ROS in the mitochondria. These ROS, in turn, could activate a regulatory protein called nuclear factor kappa B (NFκB), which controls the activities of numerous genes, including those that encode TNF–α and one of its receptors, as well as genes encoding proteins that promote apoptosis. Thus, a vicious cycle would be established in the hepatocytes: TNF–α promotes ROS production, ROS production activates NFκB, and NFκB leads to enhanced production of additional TNF– α and its receptor as well as to production of factors that promote apoptosis. This cycle eventually alters the structure of the hepatocytes, impairs their function, and can lead to hepatocyte apoptosis.

#### Transforming Growth Factor Beta (TGF–β)

The second cytokine involved in regulating apoptosis is TGF–β, generally considered an immunoregulatory cytokine. It is similar in structure and function to a large group of molecules that activate or inhibit various growth and differentiation processes. TGF–β has a range of effects, including triggering apoptosis in a variety of normal and tumor cells. For example, TGF–β, together with TNF–α, was reported to induce apoptosis in human hepatoma cells (Katz et al. 2001).

#### Consequences of Alcohol Exposure on the Effects of TGF–β

Studies have found that more TGF–β is produced in the livers of patients with alcoholic cirrhosis than in the livers of healthy people, suggesting that TGF–β might be involved in the development of alcohol-induced liver damage (Neuman et al. 2002). Moreover, this cytokine can cause the hepatocytes to produce more of certain molecules (i.e., transglutaminase and cytokeratins) that normally are responsible for giving the cells their shapes. When excess levels of these molecules are present, however, they become cross-linked to form microscopic structures called Mallory bodies, which are indicators (i.e., markers) of alcoholic hepatitis (Cameron and Neuman 1999).

TGF–β also can contribute to liver damage by activating a type of liver cell called stellate cells. In a resting state, these cells primarily serve to store fat and vitamin A in the liver. When activated, however, stellate cells produce collagen, the major component of scar tissue. Collagen production by activated stellate cells is a crucial step in the development of fibrosis in patients with alcoholic steatohepatitis. Therefore, TGF–β may be an important player in this process.

As with TNF–α, hepatocytes normally produce little or no TGF–β. Under certain conditions, however, hepatocytes can produce TGF–β or take up the cytokine from the outside (Bedossa and Paradis 1995). Alcohol might trigger the activation of TGF–β and thereby contribute to the initiation of apoptosis if this molecule enters the blood in higher concentrations (Neuman 2001; Katz et al. 2001).

## Bacterial Endotoxin, Cytokines, and Alcoholic Liver Disease

One of the factors that can enhance the production of TNF–α in macrophages, including Kupffer cells, thereby promoting apoptosis, is a bacterial protein called endotoxin or lipopolysaccharide (LPS). Endotoxin is released from the bacteria normally living in the intestine when those bacteria die. If endotoxin enters the blood and reaches the liver, it interacts with Kupffer cells, activating the cells to produce cytokines. (For more information on endotoxin and its effects, see the article in this issue by Wheeler.) Endotoxin-stimulated cytokine production may play a crucial role in the onset of liver failure during sepsis^2^ in alcoholic patients (Keshavarzian et al. 1999; Mathurin et al. 2000; Jerrells et al. 1998).

In a healthy person, endotoxin is mostly confined in the intestine and does not reach the bloodstream and the liver. Alcohol consumption, however, can lead to increased endotoxin levels in the blood by altering the permeability of the intestinal wall, allowing endotoxin to cross that wall more easily. This phenomenon, also known as “leaky gut,” was demonstrated in animal studies. In these studies, direct administration of endotoxin into the intestine led to increased endotoxin levels in the blood entering the liver in animals that had received an alcohol-containing diet, but not in animals that had not received alcohol (Neuman et al. 2002). Other animal studies found that the “leaky gut” phenomenon also occurred after a single alcohol dose and was more pronounced in animals already suffering from alcohol-induced liver injury (Mathurin et al. 2000). Researchers do not yet know, however, whether the prevalence and severity of increased gut permeability is higher in heavy drinkers with cirrhosis or severe liver injury than in heavy drinkers without alcoholic liver disease.

In the liver, endotoxin interacts primarily with Kupffer cells, and this interaction is considered crucial to the secretion of TNF–α, which then interacts with receptors on both Kupffer cells and hepatocytes, leading to the production of other inflammatory cytokines, such as IL–1, IL–6, and IL–8. The importance of this chain of events to the development or progression of alcoholic liver disease in humans was demonstrated by several observations:

- Long-term alcohol ingestion increases intestinal permeability to endotoxin, as mentioned above (Mathurin et al. 2000).
- The concentrations in the blood of TNF–α and the other inflammatory cytokines were increased in patients with alcoholic hepatitis (McClain and Cohen 1989).
- Patients with the highest cytokine concentrations in the blood had the most severe disease, as indicated by the highest rate of in-hospital mortality (McClain and Cohen 1989).
- Concentrations in the blood of both TNF–α and TNF receptors were correlated with the levels of endotoxin in the blood and with the stage of liver disease in patients with alcoholic liver disease.

Taken together, these findings indicate how important intestinally derived endotoxin and endotoxin-induced cytokines, such as TNF–α, are to the development of alcoholic hepatitis. This conclusion was further confirmed by animal experiments in which the activity of TNF–α was inhibited in different ways. For example, the animals were treated with antibodies that inactivate TNF–α, or the experiments were conducted in animals that lack the receptor for TNF–α (Molina et al. 2002). In both conditions, the animals were protected from alcohol-induced liver disease, even after long-term alcohol exposure.

Researchers have investigated the mechanisms through which endotoxin leads to TNF–α production using a mouse model in which alcohol was directly administered into the animals’ stomachs. This approach was used to compare the effects of alcohol administration in normal mice and in mice genetically engineered to lack the receptor with which endotoxin interacts on the Kupffer cells—a molecule called CD14. The study found that although alcohol administration caused liver injury in the normal mice, it had only minimal effects in the CD14-deficient mice (Yin et al. 2001). In addition, the CD14-deficient animals showed no induction of TNF–α and no activation of NFκB (which is regulated by TNF–α). These findings confirm that the binding of endotoxin to its receptors on the Kupffer cells and the resulting Kupffer cell activation and production of TNF–α are key events in the development of alcoholic liver injury. This idea is further supported by results from a recent study in human alcoholic patients, which demonstrated that a specific variant of the CD14 gene is associated with an increased risk of alcoholic liver injury (Jarvelainen et al. 2001).

Analyses in rodents also have suggested that females may be more susceptible to alcohol-induced increases in endotoxin levels in the blood than males. In one study, researchers assessed liver damage and measured the levels of the mRNAs—intermediate molecules that are formed when the genetic information encoded in a gene is “read” to produce the gene’s product—for TNF–α and other cytokines in male and female rats receiving an alcohol-containing diet. The investigators found that female rats exhibited more severe alcohol-induced liver injury than male animals (Nanji et al. 2001). In addition, the female rats had higher endotoxin levels in the blood as well as higher levels of several cytokines, including TNF–α. Finally, the female animals showed greater NFκB activation. These findings suggest that compared with males, increased endotoxin levels in the blood of females stimulate NFκB activation and cytokine production, thereby enhancing liver injury.

In summary, endotoxin seems to be an important contributor to the development of early alcoholic liver disease. In recent years, researchers have made important progress in understanding the mechanisms underlying endotoxin’s effects as well as the consequences of these effects in alcoholic liver injury. Nevertheless, several key questions remain unanswered. For example, investigators do not yet know whether endotoxin participates in advanced alcoholic liver disease and what mechanisms may underlie such an effect. Answers to these questions could provide the scientific rationale for developing and evaluating new therapeutic approaches aimed at inhibiting endotoxin’s effects in heavy drinkers with severe liver injury.

## Conclusions and Future Perspectives

Many factors acting in concert contribute to the sensitivity of liver cells to alcohol exposure and the resulting liver damage. As described in this article, cytokines have pivotal functions in many of these processes, such as the activation of the immune system that leads to chronic liver inflammation, initiation of hepatocyte apoptosis, and the effects of endotoxin. Accordingly, researchers and clinicians are trying to determine the clinical benefits that might flow from research on cytokines in the field of alcohol-induced liver disease. Some of the investigations will focus on the exact factors determining a person’s susceptibility to alcoholic liver disease. Only 20 percent of heavy drinkers actually develop liver disease (Molina et al. 2002), suggesting that some heritable factors might be involved in determining whether liver damage occurs. Other investigations should address the possibility that susceptibility to liver disease is the result of an “innate immune reactivity” to alcohol. Alternatively, in people with high susceptibility to alcoholic liver disease, liver cells may fail to sense alcohol-induced damage to neighboring cells and the resulting apoptosis-promoting signals or to remove apoptotic cells or apoptotic bodies from the liver. All of these possibilities require further investigation.

Because they have such a wide range of effects, cytokines are important targets in the management of alcoholic hepatitis and other inflammatory conditions. Once researchers fully understand how cytokines work, they may be able to develop clinical uses for cytokines and their receptors as well as for antibodies that can inhibit cytokine activity. Researchers also can generate antibodies against molecules normally found in the body (e.g., cytokines). If administered to a person or animal, these antibodies will interact with their target, thereby interfering with the activities of that molecule. For example, antibodies that interact with TNF–α may be able to inhibit the cytokine’s harmful actions, and the effectiveness and safety of these antibodies in treating patients should be evaluated. Other cytokines, such as the immunoregulatory cytokine IL–10, already are being tested in clinical trials of patients with alcoholic hepatitis. The observation that stellate cells, which are essential for the repair of damaged liver tissue (but also for the development of fibrosis), respond to the actions of the chemokine IL–8 also may lead to clinical applications.

As researchers have elucidated many of the processes occurring during apoptosis and the factors that contribute to it, they also have identified many potential points for regulating these processes that might be useful in treating alcoholic liver disease. For example, it may be possible to regulate factors influencing the induction of apoptosis or to enhance the elimination of apoptotic cells. Future research in apoptosis may provide new treatment options and increase our knowledge of the pathogenesis of human liver diseases, including alcohol-induced liver damage.

## Footnotes

1 For reviews of the metabolism of GSH, see Meister and Anderson 1983; Meister 1988.

2 Sepsis is an infection or contamination (e.g., of a wound).
